# The cancer-associated fibroblast-related signature predicts prognosis and indicates immune microenvironment infiltration in gastric cancer

**DOI:** 10.3389/fimmu.2022.951214

**Published:** 2022-07-29

**Authors:** Tsz Kin Mak, Xing Li, Huaping Huang, Kaiming Wu, Zhijian Huang, Yulong He, Changhua Zhang

**Affiliations:** ^1^ Digestive Diseases Center, The Seventh Affiliated Hospital of Sun Yat-sen University, Shenzhen, China; ^2^ Guangdong Provincial Key Laboratory of Digestive Cancer Research, The Seventh Affiliated Hospital of Sun Yat-sen University, Shenzhen, China

**Keywords:** CAFS-score, CAFs gene, Gastric cancer, immune therapy, immune microenvironment infiltration

## Abstract

**Background:**

Gastric cancer (GC) is one of the most common cancers, with a wide range of symptoms and outcomes. Cancer-associated fibroblasts (CAFs) are newly identified in the tumor microenvironment (TME) and associated with GC progression, prognosis, and treatment response. A novel CAF-associated prognostic model is urgently needed to improve treatment strategies.

**Methods:**

The detailed data of GC samples were downloaded from The Cancer Genome Atlas (TCGA), GSE62254, GSE26253, and GSE84437 datasets, then obtained 18 unique CAF-related genes from the research papers. Eight hundred eight individuals with GC were classified as TCGA or GSE84437 using consensus clustering by the selected CAF-related genes. The difference between the two subtypes revealed in this study was utilized to create the “CAF-related signature score” (CAFS-score) prognostic model and validated with the Gene Expression Omnibus (GEO) database.

**Results:**

We identified two CAF subtypes characterized by high and low CAFS-score in this study. GC patients in the low CAFS-score group had a better OS than those in the high CAFS-score group, and the cancer-related malignant pathways were more active in the high CAFS-score group, compared to the low CAFS-score group. We found that there was more early TNM stage in the low CAFS-score subgroup, while there was more advanced TNM stage in the high CAFS-score subgroup. The expression of TMB was significantly higher in the low CAFS-score subgroup than in the high CAFS-score subgroup. A low CAFS-score was linked to increased microsatellite instability-high (MSI-H), mutation load, and immunological activation. Furthermore, the CAFS-score was linked to the cancer stem cell (CSC) index as well as chemotherapeutic treatment sensitivity. The patients in the high CAFS-score subgroup had significantly higher proportions of monocytes, M2 macrophages, and resting mast cells, while plasma cells and follicular helper T cells were more abundant in the low-risk subgroup. The CAFS-score was also highly correlated with the sensitivity of chemotherapeutic drugs. The low CAFS-score group was more likely to have an immune response and respond to immunotherapy. We developed a nomogram to improve the CAFS-clinical score’s usefulness.

**Conclusion:**

The CAFS-score may have a significant role in the TME, clinicopathological characteristics, prognosis, CSC, MSI, and drug sensitivity, according to our investigation of CAFs in GC. We also analyzed the value of the CAFS-score in immune response and immunotherapy. This work provides a foundation for improving prognosis and responding to immunotherapy in patients with GC.

## Introduction

Cancer is the leading cause of premature death, which causes a huge public health and economic burden (1). According to the global cancer statistics, there were 19.3 million new cancer cases and nearly 10 million cancer-associated deaths worldwide in 2020. Among them, gastric cancer (GC) represents more than 1 million new cases and 769,000 deaths, ranking fifth in incidence (5.6%) and fourth in mortality (7.7%) (2). Cases of GC were frequently diagnosed in the advanced stage (3). Meanwhile, a trend of augmented younger GC cases (aged<50 years) also brings a severe test in therapy (2). GC is a highly molecular and phenotypic heterogeneity with a complex tumor microenvironment (TME). Research on the TME may help to explore the underlining mechanisms of tumorigenesis and development.

The TME is a heterogeneous collection of various immune cells, stromal cells, vessels, and extracellular matrix (ECM). Tumor cells and the TME act as seed and soil; the TME fosters tumor progression and mediates relapse (4). Cancer-associated fibroblasts (CAFs) are one of the most abundant cells and act as critical components among them. Activated CAFs create a conducive environment for tumorigenesis and progression. According to the research papers from PubMed, 18 CAF-related genes that were confirmed by fundamental experiments in GC were chosen for modeling purposes. Activated CAFs create a conducive environment for cancer proliferation and maintaining CSC by secreting a plethora of cytokines and chemokines, such as CXC-chemokine ligand 12 (CXCL12), interleukin-6 (IL-6), and IL-33 (5–8). Secretion of IL-6 can promote the epithelial–mesenchymal transition (EMT) and metastasis of GC *via* the JAK2/STAT3 signaling pathway. Simultaneously, IL-6 also prompts cancer immune escape by recruiting immunosuppressive cells into the TME (8, 9). Secretion of ECM-degrading proteases matrix metalloproteinases (MMPs), such as MMP11 and MMP14, directly confers a migration track by remodeling the ECM and physically pulling, promoting cancer invasion and metastasis (10–12). Besides that, CXCL12 and fibroblast growth factor 9 (FGF9) produced by CAFs facilitate tumor neovascularization to overcome a hypoxic and acidic TME (5, 7, 13). The CAF-derived hyaluronan and proteoglycan link protein 1 (HAPLN1) promotes ECM remodeling by decreasing the density and size of fibers, as well as increasing the fiber alignment, resulting in tumor invasion and aggression in GC (14, 15). Meanwhile, GC cells also release the transforming growth factor-β+ (TGFβ+) exosomes to convert mesenchymal stem cells (MSCs) into activated CAFs. The crosstalk biological aspects between CAFs and GC create a positive feedback loop to stimulate GC progression and metastasis (5). Other CAF-related genes, such as mucin 1 (MUC1), Krüppel-like Zinc-Finger Transcription Factor 5 (KLF5), tumor endothelial marker 1 (TEM1), vascular adhesion molecule 1 (VCAM1), periostin (POSTN), lysyl oxidase like 2 (LOXL2), neuropilin-2 (NRP2), rhomboid 5 homolog 2 (RHBDF2), and serum amyloid A1 (SAA1), are characterized by high expression of genes associated with a poor prognosis in patients with GC (12, 16–22). In contrast, CAF-related genes such as Sorbin and SH3 domain-containing protein 1 (SORBS1), and secreted protein acidic and rich in cysteine (SPARC) have a significantly low expression in CAF and are closely related to poor prognosis in GC (23, 24). Carcinogenesis and development are characteristic of the interaction between multiple genes and signal pathways. It is not sufficient to focus on one or two genetic biomarkers to correlate with the GC prognosis. Hence, we put up an 18-CAF-related gene subgroup classification and CAFS-score model that may provide important insights into predicting prognosis and guiding clinical practice.

In this present study, we constructed a GC scoring model (CAFS-score) based on 808 GC patients with transcriptome data and clinical information and 14 identified GC-related CAF genes, and validated its reliability with multiple datasets. We clustered those patients into two CAF subtypes according to the CAF genes’ expression levels and identified the differentially expressed genes (DEGs). Then, patients were classified into three DEG-related gene subtypes and established the CAFS-score system. The clinical practice of this scoring model was validated in GC patients, including prognosis, immune microenvironment, and drug sensitivity.

## Methods

### Dataset collection and sample information

The flowchart is described in **Supplementary Figure S1** and the samples were analyzed with staging statistics. The data of gene expression, somatic mutation, and corresponding clinical information of GC samples from The Cancer Genome Atlas (TCGA) database (https://tcga-data.nci.nih.gov/tcga/) were collected, which include tumor samples and para-cancer samples with detailed information for further analysis. In addition, 433 GC samples in South Korea (GSE84437) and 300 GC samples in the ACRG (Asian Cancer Research Group) study (GSE62254) with detailed characteristic information and survival duration were obtained from the GEO database (https://www.ncbi.nlm.nih.gov/geo/). Moreover, GSE26253 was obtained from the GEO database.

### Defining the CAF-related regulators

In the previous research study, Zang et al. (11) found that matrix metalloproteinase 11 (MMP11) secreted by CAFs is not only overexpressed in exosomes purified from plasma and GC samples, but also associated with the overall survival (OS) of GC patients. Shen et al. (15) showed that HAPLN1 is a significantly upregulated gene in CAFs of GC, and higher expression is associated with shorter OS in GC patients. CAF-derived IL-33 is upregulated in the human GC and served as a poorly prognostic marker in GC patients proved by Su et al. (6). In the research study of CAFs, Wand et al. (12) demonstrated that MMP14, LOXL2, and POSTN are characterized by high expression of genes associated with gastric tumor invasion. The previous research studies found that SORBS1, IL-6, MUC1, FGF9, KLF5, SPARC, TEM1, NRP2, CXCL12, RHBDF2, SAA1, and VCAM are significant expressions in CAF and have an association with GC (7, 8, 13, 16–24). According to our search in research papers from PubMed, we chose the 18 genes (MMP11, HAPLN1, IL-33, IL-6, SORBS1, MUC1, FGF9, KLF5, SPARC, TEM1, VCAM1, POSTN, MMP14, LOXL2, NRP2, CXCL12, RHBDF2, and SAA1) that related to CAFs in GC.

### Consensus clustering and gene clustering

According to the selected CAF-related genes, consensus clustering was utilized to identify and classify the patient into molecular subtypes by the k-means method. The “ConsensuClusterPlus” package was applied to determine the number of clusters and their stability. In addition, 1,000 repetitions were performed to ensure the stability of classification (25).

Setting the criteria of |log2(Fold Change)| > 1 and false discovery rate (FDR)< 0.05, a list of DEGs from consensus clustering was identified by utilizing the R package limma. Secondly, according to the expression of prognostic DEGs, an unsupervised clustering method was used to classify the patient into different subtype groups (Gene subtype A, Gene subtype B, and Gene subtype C) for further analysis.

To further examine the clinical value of the consensus clustering and gene clustering, we evaluated the correlations among the molecular subtypes, clinicopathological characteristics, and prognosis. The clinical characteristics included age, gender, TNM stage, and grade. Furthermore, we perform the Kaplan–Meier survival analysis in different clusters using the survival package of the R software.

### Gene set variation analysis

Gene set variation analysis is typically used to estimate variation in pathway and biological process activity in expression dataset samples (26). This method was performed to explain the differences in biological processes between two CAFs-score subtypes by using “GSVA” R packages. The gene sets of “c2.cp.kegg.v7.4.symbols.gmt” were downloaded from the MSigDB database for further GSVA. DEGs were analyzed using the R package clusterProfiler in Gene Ontology (GO) and Kyoto Encyclopedia of Genes and Genomes (KEGG),with a cutoff value of FDR< 0.05.

### Construction and validation of the prognostic model

LASSO-Cox analysis was utilized to minimize the risk of over-fitting using the “glmnet” R package. Multivariate Cox analysis was used to select the candidate genes for establishing a prognostic model (CAFS-score) in the training cohort. The CAFS-score was calculated as follows:


$$\text{CAFS}-\text{score }=\;\sum^{}\left(\text{Expi }\times \text{ coefi}\right)$$

where Coefi and Expi denote the risk coefficient and expression of each gene, respectively. The cutoff point was determined using the “survminer” package. According to the CAFS-score, we revealed that the survival curve was used for visualization with both training and testing cohorts in the high- or low-risk group by Kaplan–Meier analysis. *p*-values< 0.05 were considered to be statistically significant.

### Clinical correlation and stratification analyses of the CAFS-score

Between the risk score and clinicopathological variables, univariate and multivariate Cox regression analysis was done to validate whether the CAFS-score is an independent prognostic predictor. The results were revealed in the forest map. Thorsson et al. (27) found that all tumors could be divided into six immune subtypes, namely, wound healing (C1), IFN-γ dominant (C2), inflammatory (C3), lymphocyte depleted (C4), immunologically quiet (C5), and TGF-β dominant (C6). Therefore, we performed the factor of immune sub-type (https://tcga-pancan-atlas-hub.s3.us-east-1.amazonaws.com/download/Subtype_Immune_Model_Based.txt.gz) between different risk groups, using the R software of “RColorBrewer”.

For the gene mutation analysis, information on genetic alteration was downloaded from the TCGA and GEO databases. The R package “Maftools” was utilized for analyzing the gene mutation in different risk subgroups. Moreover, the correlation between the CAFS-score and total mutation burden (TMB) was analyzed and performed in our study. Further analysis, we revealed the relationship between the CAFS-score and CSC index. The CSC index was calculated by using innovative one-class logistic regression (OCLR) machine-learning algorithm (28). In addition, we explored the relationship between the different risk groups and MSI.

### Identification of immune characteristics for the CAFS-score

CIBERSORT (https://cibersort.stanford.edu/) is a common algorithm to obtain cell composition from solid tumors or gene expression profiles, which was used to analyze the enrichment of immune cells in the CAFS-score for our study. The different content of immune infiltrating cells between the high- and low-risk groups was analyzed by Wilcoxon signed rank test and performed on the box chart for the TCGA cohort. In further analysis, we showed the correlations between the abundance of immune cells and four genes in the prognostic model according to the training cohort.

### Assessment of immunotherapy

For the predicted assessment of the patient with immunotherapy in the prognostic value of the CAFS-score, the time-dependent receiver operating characteristic (ROC) curve analysis was performed for obtaining the area under the curve (AUC). In addition, we not only downloaded the tumor immune dysfunction and exclusion (TIDE) score online (http://tide.dfci.harvard.edu/) but obtained the T-cell-inflamed signature (TIS) score calculating the average value of a log2-scale normalized expression in the 18 signature genes (29). Thereafter, we revealed the results after comparing the prognostic values of the CAFS-score, TIDE, and TIS by using the R package “timeROC” and performed time‐dependent ROC curve analyses to obtain the AUC.

Besides comparing the prognostic values of the CAFS-score, TIDE, and TIS, we also utilized the immunophenoscore (IPS) to predict the response of immune checkpoint inhibitors (ICIs) based on the expression of the main component in tumor immunity. According to a scale with a range of 0–10 based on representative cell-type gene expression *z*-scores, IPS was calculated where the immunogenicity was positively correlated with the score of IPS (30). The IPSs of patients with GC were derived from The Cancer Immunome Atlas (TCIA) (http://tcia.at/home). The result was performed using the R package “ggpubr”.

### Assessment of drug sensitivity

The sensitivity of various drugs was predicted in patients between two CAFS-score subgroups. The pRRophetic R package was utilized for drug prediction (31). Wilcoxon signed-rank test was utilized to explore the difference in IC_50_ between different risk groups. The results were performed by using the R package “ggplot2”.

### Establishment and validation of a nomogram scoring system

According to the independent prognosis outcome, a predictive nomogram was produced by the clinical characteristics and the CAFS-score using the “rms” package of R. In the nomogram scoring system, each variable has a corresponding score, and the total score is obtained by adding up the scores of all variables for each sample (32). The nomogram was evaluated using ROC curves for the 1-, 3-, and 5-year survival rates. The nomogram calibration plots were used to describe the predictive value of the anticipated 1-, 3,- and 5-year survival events in relation to the actual observed outcomes.

### Statistical analysis

R software and R Bioconductor packages were used for the data analysis.(version 4.1.2; https://www.R-project.org). Comparison of non-parametric or parametric method differences was carried out using Wilcoxon test, Kruskal–Wallis test, and *t*-test or one-way ANOVA. Spearman’s and distance correlation analyses were used to calculate the correlation coefficients. The validity of the model was verified by the receiver operating characteristic (ROC) curve. Based on the correlation between the CAFS-score and patient survival, the best cutoff point of survival information for each cohort was determined by the Survminer package. Kaplan–Meier test and Log-rank test were used to analyze the prognosis of survival curve, which were used to assess differences between groups. The hazard ratio (HR) of CAF regulators and CAF-related genes was computed by using the univariate Cox regression model. To verify whether the CAFS-score was an independent prognostic predictor, we incorporated the CAFS-score and CAF-related clinical parameters into a multivariate Cox regression model analysis. All statistical analyses were bilateral, and statistical significance was set at *p*< 0.05.

## Result

### Overview of genetic changes and expression variations of CAF-related regulators in GC

**Figure 1:**
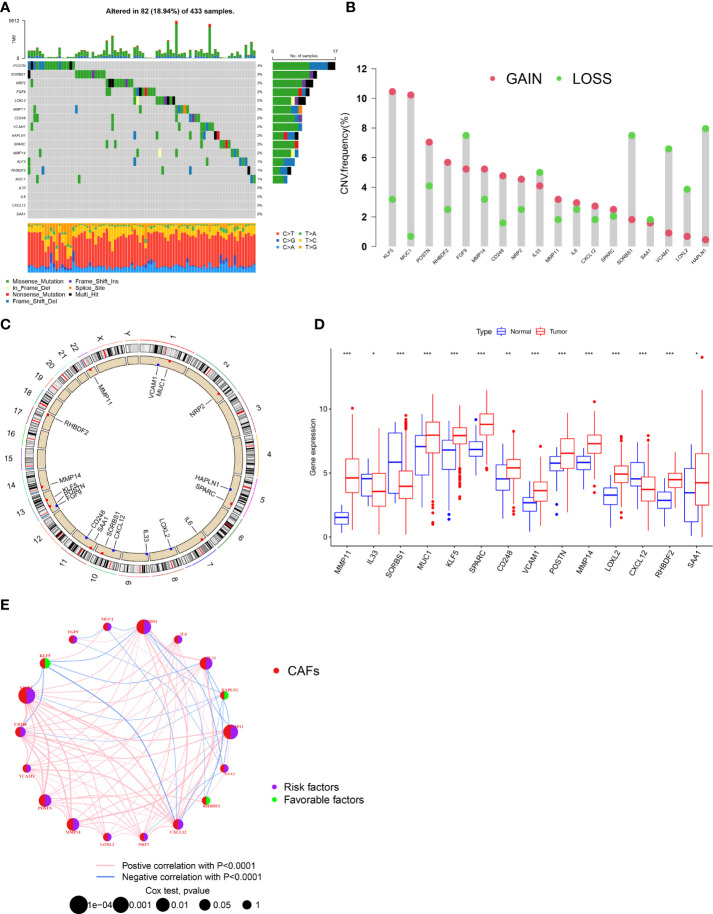
Genetic and transcriptional alterations of CAFs-related genes in GC. **(A)** Mutation frequencies of 18 CAFs-related genes in 433 patients with STAD from the TCGA cohort. **(B)** Frequencies of CNV gain, loss, and non-CNV among CAFs-related genes. **(C)** Locations of CNV alterations in CAFs-related genes on 23 chromosomes. **(D)** Expression distributions of 1 8 CAFs-related genes in normal and GC tissues. **(E)** Interactions among CAFs-related genes in GC. The connecting line among CAFs-related genes indicates their interaction, and the thickness of lines represents the strength of the association between CAFs-related genes. Blue and pink represent negative and positive correlations, respectively. *P<0.05, **P<0.01, ***P<0.001.

First, we analyzed the gene mutations to understand the mutation types of the selected CAF-related genes in GC samples (**Figure 1A**). At the genetic level, CAF-related regulator mutations were found in 82 of the 433 samples (approximately 18.94%). The study revealed that POSTN had the highest frequency of mutations. In contrast, we observed that IL-33, IL-6, CXCL12, and SAA1 do not have any mutations in any GC samples. We determined the frequency of copy number variants (CNVs) in selected CAF-related genes and discovered changes in selected CAF-related genes with CNVs on the chromosome (**Figures 1B, C**). For example, KLF5 was shown to be a frequent modification, with the majority of the changes focusing on copy number amplification on the 13 chromosomes. In terms of expression levels, 14 of 18 selected genes in tumor samples showed a significant difference as compared with normal samples (**Figure 1D**). A network was created to show the whole landscape of the selected genes’ interconnections, regulator linkages, and prognostic significance in patients with GC (**Figure 1E**).

### Identification of CAF subtypes in GC

**Figure 2:**
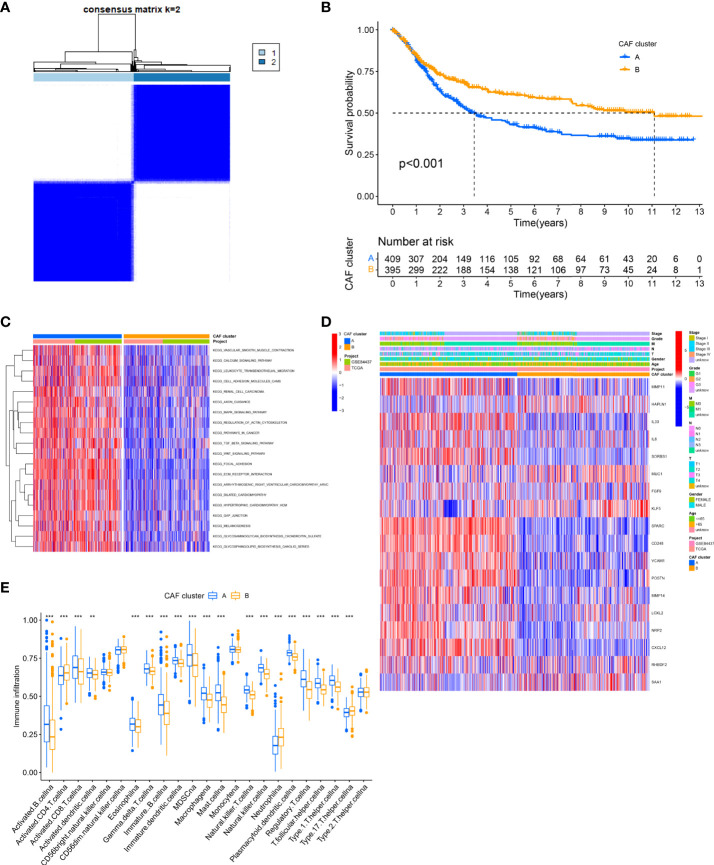
Identification of CAFs subtypes in GC. **(A)** Consensus matrix heatmap defining two clusters (k = 2) and their correlation area. **(B)** Univariate analysis indicating 18 CAFs-related genes corelated with the OS time. **(C)** GSVA of biological pathways between two distinct subtypes. (Red and blue represent activated and inhibited pathways, respectively). **(D)** Differences in clinicopathologic features and expression levels of CAFs-related genes between the two distinct subtypes. **(E)** The 22 infiltrating immune cell types in the two GC subtypes. **P<0.01, ***P<0.001.

First, we analyzed and revealed the selected CAF genes of prognostic value in the 808 GC patients using the univariate Cox regression and Kaplan–Meier analysis (**Figure S2**). We used the unsupervised clustering technique to identify different regulatory patterns based on the expression levels of 18 CAF-related regulators. For classifying the entire cohort into subtypes A (*n* = 444) and B (*n* = 364), the result revealed that k = 2 seems to be the perfect choice (**Figure 2A** and **Table S1**). For the survival analysis, the results showed that cluster B had a better survival probability than cluster A (**Figure 2B**). Moreover, the variations in biological behavior between these two patterns were investigated using gene set variation analysis (GSVA) enrichment analysis (**Figure 2C** and **Table S2**). It showed that cluster A was enriched in terms of pathways associated with ECM and tumor invasion, including the ECM–receptor interaction and Focal adhesion. **Figure 2D** illustrates that the CAF gene subtype B patterns were also linked to advanced TNM stages, particularly the T stage. We explored the 22 infiltrating immune cell types in the two GC subtypes (**Figure 2E**). The result showed that most of the infiltrating immune cells were significantly different between the two GC subtypes, except CD56 bright natural killer cells, CD56 dim natural killer cells, monocytes, and Type 2 T helper cells. In addition, infiltrating immune cells were abundant in cluster A, except activated CD4 T cells, neutrophils, and Type 17 T helper cells. Following this, we confirmed that the 18 CAF-related regulators could be used to discriminate the two regulatory patterns (**Figure S3A**).

Setting the criteria of |log2(Fold Change)| > 1 and FDR< 0.05, 342 DEGs from consensus clustering were identified. Under the functional enrichment analysis, GO analysis and KEGG pathway analysis were performed, significantly related to the DEGs. For **Figure S3B**, a total of 342 DEGs were significantly associated with 789 GO terms (details in **Table S3**), such as ECM organization for Biological Process (BP), collagen-containing ECM for Cellular Component (CC), and ECM structural constituent for Molecular Function (MF). In addition, the result of the top 18 KEGG pathways associated with candidate genes is illustrated in **Figure S3C**, such as PI3K-Akt signaling pathway, Focal adhesion, and Protein digestion and absorption. The results of GO term and KEGG suggested that the CAFs play a dynamic role in the ECM and tumor invasion.

### Identification of gene subtypes based on DEGs

**Figure 3:**
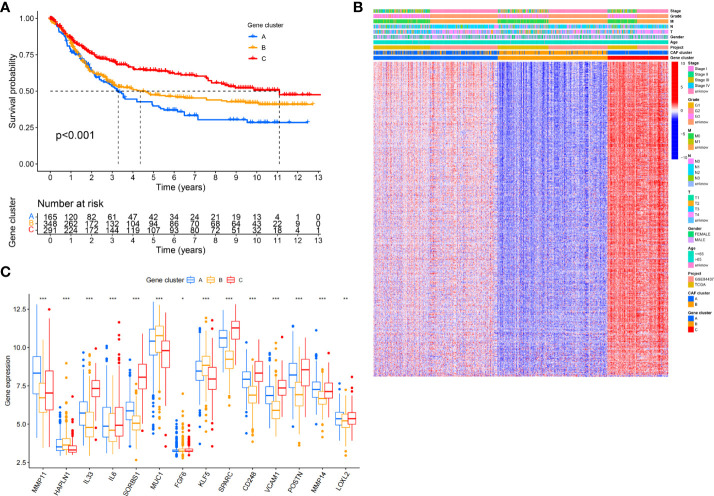
Identification of gene subtypes based on DEGs. **(A)** Kaplan-Meier curves for RFS of the three gene subtypes (log-rank tests, p < 0.001). **(B)** Relationships between clinicopathologic features and the three gene subtypes. **(C)** Differences in the expression of 18 CAFs-related genes among the three gene subtypes. *P<0.05, **P<0.01, ***P<0.001.

After further analysis of univariate Cox regression in DEGs, we identified 316 genes related to survival time (*p*< 0.05), which were used in further analysis. A consensus clustering technique was utilized to classify patients into three genomic subgroups based on prognostic genes, termed gene subtypes A to C, to further validate this regulatory mechanism. According to Kaplan–Meier curves, patients in subtype A had the worst survival, whereas patients in cluster C had a favorable survival time (**Figure 3A**). Furthermore, the gene subtype A pattern was linked to an advanced TNM stage (**Figure 3B**). Expression of 14 of the previous18 selected CAF-related genes had a significant difference among the three gene subtypes, as expected based on the patterns (**Figure 3C**).

### Establishment risk assessment model and survival outcomes in GC

**Figure 4:**
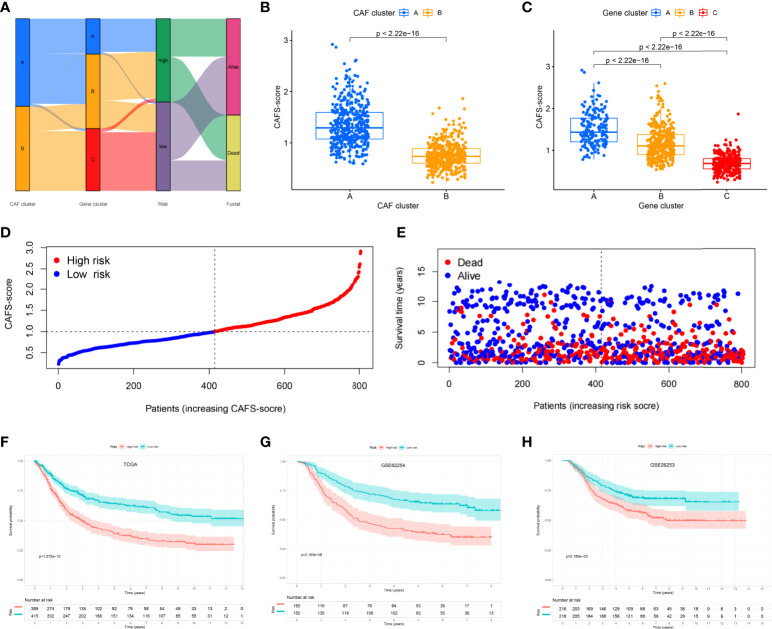
Establishment risk assessment model and survival outcomes in GC. **(A)** Alluvial diagram of the subtype distributions in groups with different CAFS-score and survival outcomes. **(B)** Differences in CAFS-score between two CAF subtypes. **(C)** Differences in CAFS-score between three gene subtypes. **(D, E)** Ranked dot and scatter plots representing the CAFS-score distribution and patient survival status. **(F-H)** Kaplan-Meier analysis of the RFS between the two risk groups in the TCGA, GSE62254, and GSE26253 cohort.

The CAFS-score was constructed using DEGs connected to subtypes. The distribution of patients in the two CAFs subtypes, three gene subtypes, and two CAFS-score groups is revealed in **Figure 4A**. According to the least partial likelihood deviance, 10 OS-associated genes remained after LASSO regression analysis (**Figures S4A, B**). This was followed by multivariate Cox regression analysis, wherein four genes (MMP11, HEYL, NNMT, and PDK) were eventually obtained to construct the prognostic model, named the “CAFS-score”. Based on the results of the multivariate Cox regression analysis, the CAFS-score was constructed as follows:


Risk score=expression level of MMP11∗(0.13641)+expression level of HEYL∗(0.13075)+expression level of NNMT∗(0.11341)+expression level of PDK4∗(0.12228)

After further analysis of applying risk score, there was a significant difference in the CAFS-score between CAF subtypes and gene subtypes (**Figures 4B, C**). The distribution plot of the CAFS-score demonstrated that the survival times were reduced while the CAFS-score increased (**Figures 4D, E**). Finally, we used the risk score to re-distinguish high- and low-risk groups in the training cohort and testing cohort. As illustrated in **Figures 4F, G**, low-risk patients had a better OS than high-risk patients (*p*< 0.05, log-rank test) whether in the training cohort or the GSE62254 cohort. Consistent with the results of the training cohort, patients from the low-risk group had a better OS than high-risk patients (*p*< 0.05, log-rank test) in the GSE26253 cohort (**Figure 4H**).

### Clinicopathologic characteristics of TCGA in the CAFS-score

**Figure 5:**
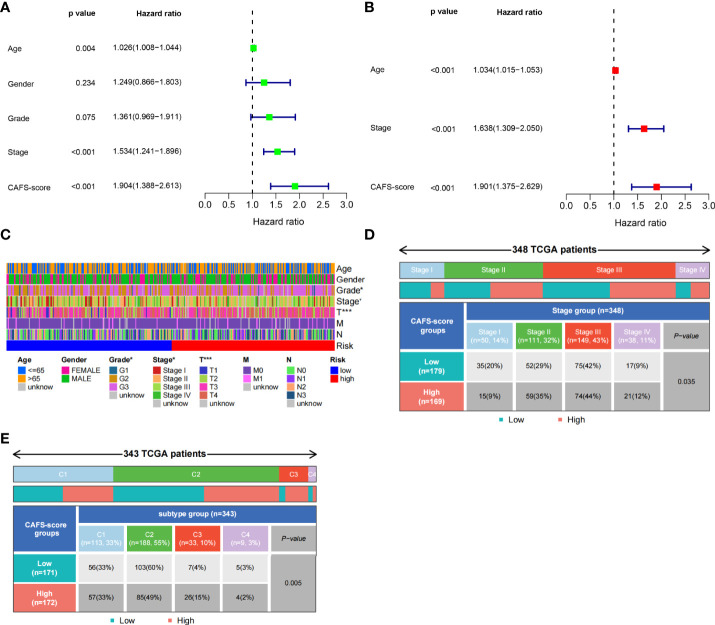
Clinicopathologic characteristics of TCGA in CAFS-score. **(A)** The Univariate Cox regression analysis in CAFS-score subgroups. **(B)** The multiple Cox regression analysis in CAFS-score subgroups. **(C)** The clinicopathologic characteristics of GC patients in the TCGA cohort. **(D, E)** The staging and the immune subtypes was significantly related to the risk between the two CAFS-score subgroups, respectively. *P<0.05, ***P<0.001.

Based on univariate Cox regression analysis, **Figure 5A** illustrates that age, CAFS-score, and stage were significantly associated with the prognosis of GC. After further multivariate Cox regression analysis, **Figure 5B** shows that the CAFS-score presented as an independent prognostic factor after adjusting for other clinicopathologic factors. The clinicopathologic characteristics of GC patients in the TCGA cohort are shown in **Figure 5C**, which revealed a significant difference in grade and TNM stage, especially for T stage. Furthermore, we found that there was more early TNM stage in the low-risk subgroup, while there was more advanced TNM stage in the high-risk subgroup (**Figure 5D**, *p*< 0.05). In addition, we found that the immune sub-types were significantly related to the risk between the two risk subgroups (**Figure 5E**, *p*< 0.05). Meanwhile, GSVA enrichment analysis was used to explore the differences in biological behavior between the two risk subgroups (**Figure S5**). It illustrated that the high-risk groups were associated with ECM and tumor invasion, including the ECM–receptor interaction and Focal adhesion.

### Relationship of the CAFS-score with TMB, MSI, and CSC index

**Figure 6:**
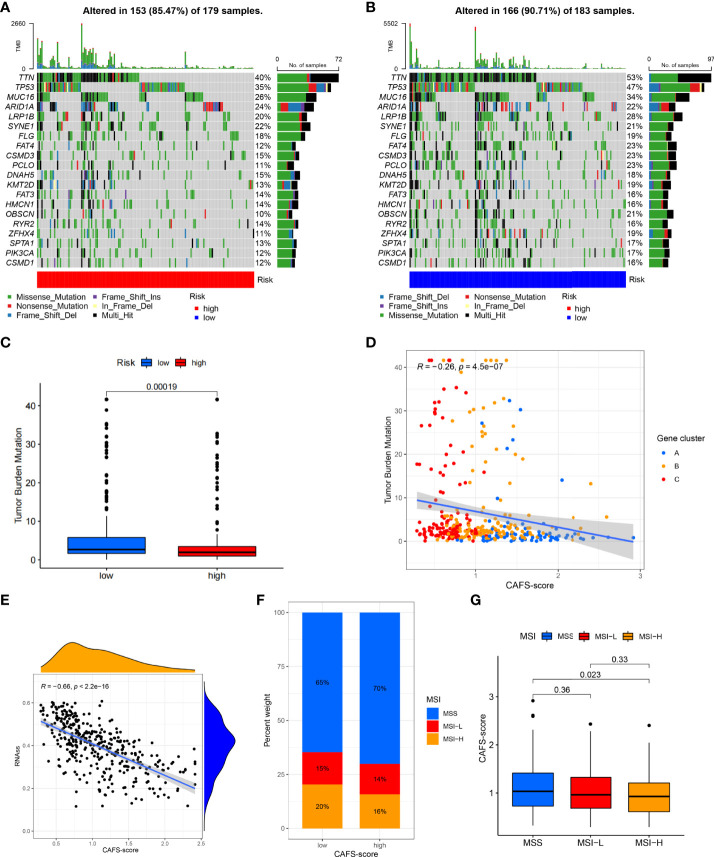
Characteristic in gene mutation and relationship of CAFS-score with MSI and CSC index. **(A, B)** Significantly mutated genes in the mutated GC samples of the high and the low risk groups, respectively. Mutated genes (rows, top 20) are ordered by mutation rate; samples (columns) are arranged to emphasize mutual exclusivity among mutations. The right shows mutation percentage, and the top shows the overall number of mutations. The color-coding indicates the mutation type. **(C)** The TMB of two differen risk subgroups. **(D)** Relationships between CAFS-score and TMR in three gene subtypes. **(E)** Relationships between CAFS-score and CSC index. **(F, G)** Relationships between CAFS-score and MSI.

We analyzed the gene mutations to further understand the immunological nature in different risk subgroups. We identified the top 20 genes with the highest mutation rates in the high-risk subgroup (**Figure 6A**) and low-risk subgroup (**Figure 6B**). The results illustrated that missense mutation was the most common mutation type. The mutation rates of TTN, MUC16, and TP53 were not only higher than 25% in both groups, but the most common in both groups. Moreover, we analyzed the relationship between the risk score and TMB. The expression of TMB was significantly higher in the low-risk subgroup than in the high-risk subgroup (**Figure 6C**). In addition, the risk score was correlated with TMB in gene subtypes (*r* = −0.26, *p*< 0.05), as revealed in **Figure 6D**.

Moreover, we observed that the risk score was correlated with the CSC index (*r* = −0.66, *p*< 0.05), as shown in **Figure 6E**. Finally, we revealed that a low CAFS-score was linked to MSI-H status, whereas a high CAFS-score was linked to microsatellite stable (MSS) status (**Figures 6F, G**).

### Immune infiltration in CAFS-score subgroup

**Figure 7:**
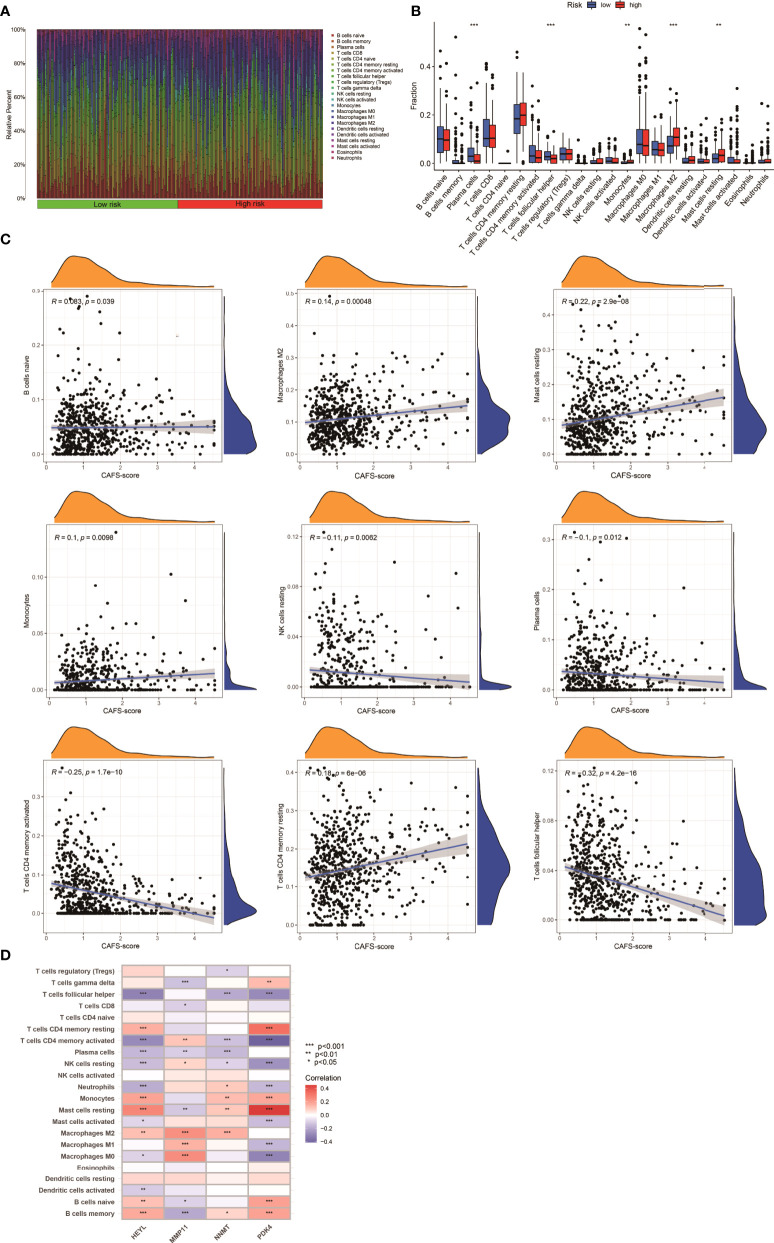
Immune Infiltration in two CAFS-score subgroup (TCGA). **(A)** Composition of immune cells in two CAFS-score subgroup. **(B)** The Relative immune infiltration score of 22 immune cells between low- and high-risk groups. **(C)** Relationships between CAFS-score and different immune cells. **(D)** Correlations between the abundance of immune cells and four genes in the proposed model. *P<0.05, **P<0.01, ***P<0.001

The gene expression matrix of the TCGA database in GC was uploaded into CIBERSORT web to estimate the fractions of 22 immune cells. Next, we explored the composition of immune cells in different risk subgroups (**Figure 7A**) in the TCGA database of GC samples. The result illustrated that the patients in the high-risk subgroup had significantly higher proportions of monocytes, M2 macrophages, and resting mast cells, while plasma cells and follicular helper T cells were more abundant in the low-risk subgroup (*p*< 0.05) (**Figure 7B**). We also found that the infiltrating abundance of M0 macrophages, resting mast cells, resting dendritic cells, M2 macrophages, resting NK cells, and CD8 T cells was significantly related to OS (*p*< 0.05) (**Figure S6**). The higher infiltrating abundance of macrophages M2 was associated with poorer OS.

Based on the training set, we explored that the CAFS-score was positively correlated to naïve B cells, M2 macrophages, resting mast cells, monocytes, and CD4 memory resting T cells (**Figure 7C**). The four genes were also shown to be highly linked to the majority of immune cells (**Figure 7D**). Therefore, the CAFS-score is statistically correlated with the infiltration of most kinds of immune cells. This means that the CAFS-score has the potential to indicate poor prognosis under different immune infiltrations.

### Immunotherapy prediction

**Figure 8:**
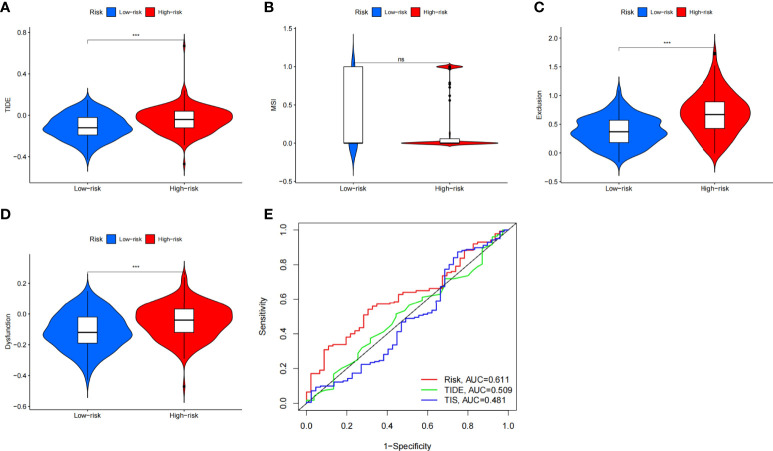
The prognostic value of CAFS-score in immunotherapy from TCGA cohort. **(A–D)** TIDE, MSI, T cell exclusion, and T cell dysfunction score in two CAFS-score subgroup, respectively. **(E)** ROC analysis of CAFS-score, TIDE, and TIS on OS in GC cohort. ***P<0.001, ns, P>0.05.

This study aims to assess the potential efficacy of immunotherapy in a clinical setting in different risk subgroups. To illustrate, a higher TIDE prediction score corresponded with a higher potential for immune evasion, which proved that the patients were unlikely to benefit from the treatment of immunotherapy. The subgroup with low risk had lower TIDE scores than the subgroup with high risk, which means that patients with low risk were more likely to benefit from ICI treatment than those with high risk (**Figure 8A**), whereas higher TIDE prediction scores are associated with poorer benefits from ICI treatment. For a lower TIDE score, the patients with low risk might have a better prognosis than those with high risk. Moreover, we found that the T-cell exclusion score (**Figure 8C**) and T-cell dysfunction (**Figure 8D**) were significantly different between the two risk subgroups, except the MSI score (**Figure 8B**). Under the AUC, the result illustrated that our risk model was the best compared with TIS and TIDE (**Figure 8E**). Therefore, we suggested that the predictive value of risk was comparable with 18-gene TIS and TIDE.

**Figure 9:**
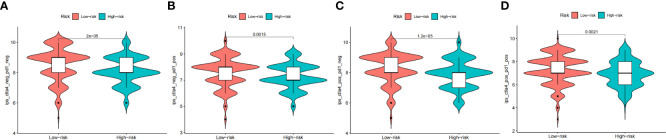
The prognostic value of CAFS-score in immunotherapy from TCGA cohort. **(A–D)** The vioplot of the difference expression of CTLA4 and PD-1 between high- and low-risk groups.

Besides utilizing the TIDE score, we also analyzed the correlation between the risk and IPS in GC patients to predict the response of ICIs. For the IPS, cytotoxic T lymphocyte antigen-4 (CTLA-4), programmed cell death protein 1 (PD-1), and programmed death ligand-1 (PD-L1) were the immune checkpoints. Therefore, their immune checkpoints were utilized to evaluate the potential ICI treatment (**Figure 9**). As a result, we found that they were significantly elevated in the low-risk group, which was categorized by the risk, which means more immunogenicity on ICIs in the low-risk group. Collectively, these results suggested that the low-risk group was more likely to have an immune response and respond to immunotherapy.

### Drug sensitivity

**Figure 10:**
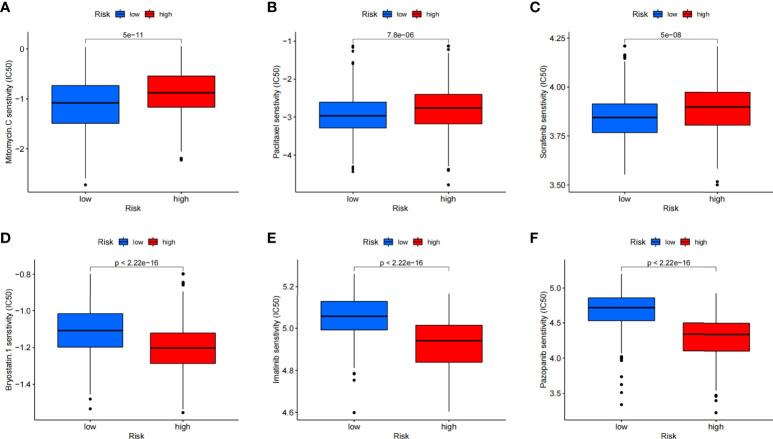
Relationships between CAFS-score and medicine sensitivity. Lower IC50 of indicated chemo-therapeutics drugs in low **(A–C)** and high **(D–F)** CAFS-score group, respectively.

Except for assessment of ICI treatment, we tried to find the links between different risk groupings and the effectiveness of chemotherapy for treating GC in the training cohort. We illustrated that the low risk was associated with a lower half inhibitory concentration (IC_50_) of chemo-therapeutics such as Mitomycin C, Paclitaxel, and Sorafenib (*p*< 0.05), whereas the high risk was associated with a low IC_50_ such as Pazopanib, Imatinib, and Bryostatin (*p*< 0.05). Therefore, **Figure 10** illustrates that the CAFS-score acted as a potential predictor for chemo-sensitivity, and details are shown in **Table S4**.

### Establishment of a nomogram to predict survival

**Figure 11:**
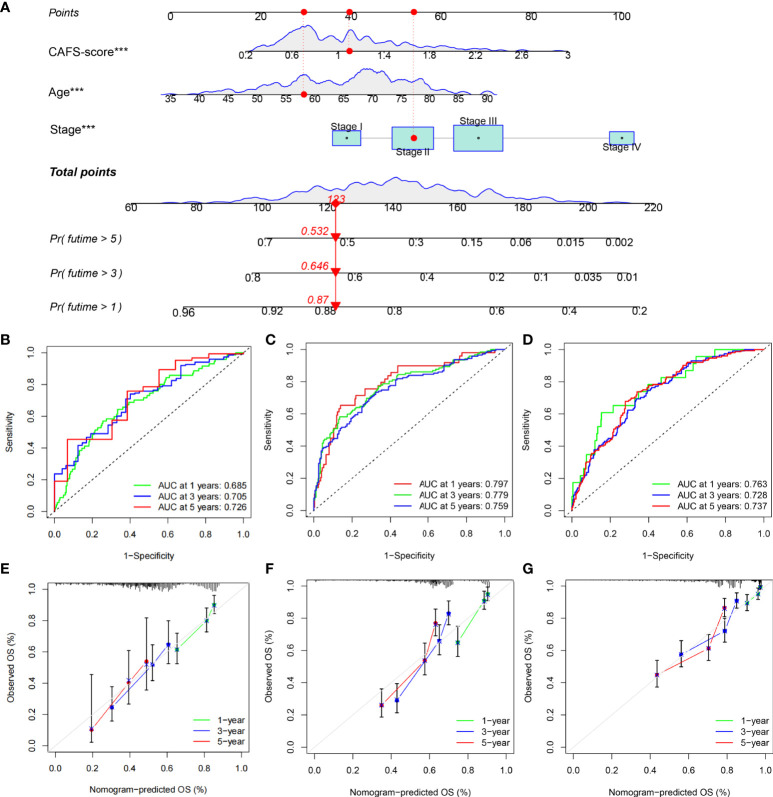
Construction and validation of a nomogram. **(A)** Nomogram for predicting the 1-, 3-, and 5-year OS of GC patients in TCGA cohort. **(B-D)** ROC curves for predicting the 1-, 3-, and 5-year ROC curves in TCGA, GSE62254, and GSE26253 cohorts. **(E-G)** Calibration curves of the nomogram for predicting of 1-, 3-, and 5-year OS in the TCGA, GSE62254, and GSE26253 cohorts. ***P<0.001.

Given the inconvenient clinical value of the CAFS-score in predicting OS in patients with GC, a nomogram incorporating the CAFS-score and clinicopathological characteristics was developed to predict 1-, 3-, and 5-year OS rates in patients with GC (**Figure 11A**). For the TCGA, GSE62254, and GSE26253 cohorts, our AUC studies on the nomogram model revealed a good accuracy for OS at 1, 3, and 5 years (**Figures 11B–D**). In the TCGA, GSE62254, and GSE26253 cohorts, the proposed nomogram performed similarly to an ideal model according to the calibration plots (**Figures 11E–G**). Finally, we compared the nomogram’s prediction accuracy to that of the TNM stage in the TCGA, GSE62254, and GSE26253 cohorts (**Figure S7**). The results illustrated that the nomogram’s AUC values were greater than the TNM stage in three cohorts.

## Discussion

Globally, GC is one of the leading causes of preventable death and ranks fifth in incidence (5.6%) and fourth in mortality (7.7%) among malignant tumors (2, 33). The etiology of this tumor remains poorly understood. Despite the rapid development of biological agents, the choices of treatment in GC are limited until now. Meanwhile, the prognosis of advanced GC under the primary treatment remains disappointing (34). CAFs are the most abundant cell in the TME of GC. By exerting ECM deposition and remodeling, the activated CAFs exhibit extensive reciprocal signaling interaction, crosstalk with immune cells, and mediate oncogenesis and progression of GC (5, 9, 35). However, it is not precise to focus on a single gene or an entire CAF-related gene set to correlate with GC prognosis. Hence, the results of the present study are based on an 18-identified GC-related CAF gene set and constructed a CAFS-risk score to predict prognosis and guidelines for the individualized clinical strategies of GC.

Consensus clustering algorithms offer the ability to efficiently analyze and identify clusters of patients with different characteristics in a large amount of data (36). Therefore, we used this unsupervised algorithm to identify two distinct molecular subtypes based on the expression levels of 18 CAF-related regulators. We found that patients with subtype B had a better survival probability than subtype A patients. We also used GSVA enrichment analysis to investigate the variations in biological behavior between these two subtypes. Subtype A was enriched in terms of pathways associated with ECM and tumor invasion, especially ECM–receptor interaction and Focal adhesion. Some literature proved that the ECM receptor contributes to GC progression and poor survival (37). Focal adhesion-related proteins independently predicted the poor clinical prognosis of GC (38). Moreover, this consensus clustering algorithm was also used to classify the patient into three different subtype groups for deeper analysis according to the expression of prognostic DEGs.

In this study, we constructed the powerfully effective prognostic model and demonstrated its predictive ability. The expression levels of four genes (MMP11, PDK4, HEYL, and NNMT), including the CAF-related gene, MMP11, were also explored in GC. MMP11, one kind of ECM-degrading protease, in exosomes was secreted from CAFs and promoted GC cell migration and invasion by regulating and shaping the TME. Normally, MMP11 is absent in human organs, and the expression level of MMP11 correlates to the OS of gastric patients (10, 11). HEYL and NNMT are usually upregulated in GC. Both act as an oncogenic factor to promote the carcinogenic and progressive process of GC *via* activating CDH11 and transforming growth factor-β (TGF-β) expression, respectively (39, 40). PDK4 promotes the Warburg effect in GC and the overexpression of PDK4 also leads to drug resistance and GC metastasis (41).

To further improve the accuracy of prognostic prediction, we constructed and validated a nomogram by screening various indexes, CAFS-score, age, gender, and pathological stage. The result illustrated that age, CAFS-score, and pathological stage were significantly associated with the prognosis of GC. Under the newest edition of AJCC, In et al. found that the pathological stage was closely associated with the prognosis of GC (42, 43). Moreover, we developed a quantitative nomogram that increased performance and made it easier to use the CAFS-score. GC is considered as an age-related disease, because older cancer patients have been shown to have poorer OS outcomes (44). According to the result, we found that the CAFS-score presented as an independent prognostic factor. Thorsson et al. (27) found that all tumors could be divided into six immune subtypes that are intended to serve as a resource for future targeted studies to further advance the field. Therefore, we found that the factor of immune subtypes was closely correlated with the risk score. These immune subtypes represent features of the TME that largely cut across traditional cancer classifications to create subgroups and suggest that certain treatments may be independent of histological type (27).

Numerous studies on various tumors have shown that patients with high TMB tend to favor good survival rates (45). Similarly, we illustrated that higher TMB was seen in the low risk of the CAFS-score. It means that high TMB has significantly better OS than the patients with a low TMB. In some literature, MUC16 mutations are associated with better prognosis and higher TMB in GC, while TTN mutations are associated with better response to immune checkpoint blockade in solid tumors (43, 46, 47). Even though TP53 is one of the most frequently mutated genes, it is insufficient to properly predict patient outcomes (48, 49). Patients with a high level of MSI respond better to immunotherapy and may benefit from it (50). Therefore, GC patients with a low-risk score had a better benefit from immunotherapy. In addition, GC cells with a lower CAFS-score exhibited more pronounced stem cell characteristics and a lower degree of cell differentiation.

To explore the importance of immune cell infiltration in GC with different risk groups for our study, CIBERSORT was utilized for analyzing the relative proportion of 22 immune cells in each GC specimen. As we know, circulating monocytes in peripheral blood migrate to tissue where they differentiate to macrophages or dendritic cells. Macrophages can be differentiated into two main types (M1 macrophages and M2 macrophages) depending on mode of activation and function. Meanwhile, some literature indicated that M2 macrophages can promote tumor growth in GC (51, 52). Consistent with these studies, we illustrated that less infiltration of M2 had a better prognosis. Wang et al. analyzed that the greater risk score resulted in a considerably shorter total survival time, and there was a positive association between risk score and dendritic cell infiltration in GC (53). Our result showed that the CAFs gene is associated with ECM-associated pathways. Therefore, less infiltration of dendritic cells had a better prognosis according to our result. The literature revealed that infiltrating mast cells are seen in large numbers in GC, which is linked to tumor growth and predicts poorer OS (54). Consistent with this study, we illustrated that more infiltration of mast cells had a poorer prognosis. According to the evidence, we believed that the CAFS-score had the potential to reflect immune cell infiltration as well as the prognostic significance of various immune cell types.

In our study, we explored the CAFS‐based differences in the TME that might reflect different immune benefits from ICI therapy by utilizing TIDE and IPS. Firstly, the TIDE score is associated with the two different mechanisms of immune escape, namely, dysfunction of tumor-infiltrating cytotoxic T lymphocytes (CTLs) and exclusion of CTL. For the evidence, TIDE scores correlate to the potential of anti-tumor immune escape and thus show the response rate to ICI treatment (55). According to our analysis, we found that the lower CAFS-score corresponds to a lower score of the TIDE than high CAFS-score patients, and thus higher ICI response might predictably occur. Secondly, the IPS is mainly associated with a couple of immune checkpoints, including CTLA-4, PD-1, and PD-L1. For the clinical trial with immunotherapy, literature demonstrated that avelumab (anti-PD-1) has anti-tumor activity and is safe for patients with GC, which is administered as maintenance therapy (after the disease is under control with standard chemotherapy) (56). Consistent with our results, it was significantly higher in the low-risk group, which was categorized by the CAFS-score. Based on the results identified with TIDE and IPS, we discovered that the CAFS-score may distinguish between various outcomes in individuals receiving immunotherapy. The CAFS-predictive score’s value has the potential to offer a theoretical foundation for ICI treatment selection in clinical trials. This predictive model could assist to speed up the development of personalized cancer therapies.

According to the clinical trial, the literature showed that immune therapy in patients with GC had a great outcome before the disease was under control by standard chemotherapy (56). We wanted to figure out if the combination treatment with chemotherapy and immune therapy in GC had a better efficacy for further study. Therefore, we explored the sensitivity of various drugs in patients between two risk subgroups. Our study demonstrated that the low-risk group had a high potential for ICI response; meanwhile, we found out that the low risk was highly associated with sensitive drugs, including Mitomycin C, Paclitaxel, and Sorafenib. It means that further studies can focus on the combined treatment for GC patients. These drugs were found by the predictive model of the CAFS-score and had potential to treat GC under specific conditions (57–59).

Our comprehensive analysis demonstrated that the CAFS-score grouping might help to differentiate the clinicopathological features, immune infiltration, and clinical prognosis of GC patients. Furthermore, this study sheds light on the role of the CAFS-score in prognosis predictive value, and provides insights into individualized strategies, guiding immunotherapy, and chemotherapy. However, further studies on interactions among these model genes and their biological mechanisms are needed.

## Data availability statement

The datasets presented in this study can be found in online repositories. The names of the repository/repositories and accession number(s) can be found in the article/**supplementary material**.

## Author contributions

Conceptualization: TM, XL, HH, and ZH. Data curation: TM, XL, HH, and KW. Formal analysis: TM, XL, HH, and KW. Data analysis: TM and XL. Investigation: TM, XL, and HH. Methodology: TM, XL, and HH. Project administration: CZ, YH, and ZH. Resources: TM, XL, and HH. Original draft: TM, XL, and HH. Writing, review, and editing: TM, XL, HH, and ZH. All authors contributed to the article and approved the submitted manuscript.

## Funding

This study was supported by the Guangdong Provincial Key Laboratory of Digestive Cancer Research (No. 2021B1212040006), the Shenzhen Sustainable Project (KCXFZ202002011010593), the National Natural Science Foundation of China (82073148), the National Natural Science Foundation of China (81772579), the Sanming Project of Medicine in Shenzhen (No. SZSM201911010), the Shenzhen Key Medical Discipline Construction Fund (No.SZXK016), and the Guangdong–Hong Kong–Macau University Joint Laboratory of Digestive Cancer Research (2021LSYS003).

## Conflict of Interest

The authors declare that the research was conducted in the absence of any commercial or financial relationships that could be construed as a potential conflict of interest.

## Publisher’s note

All claims expressed in this article are solely those of the authors and do not necessarily represent those of their affiliated organizations, or those of the publisher, the editors and the reviewers. Any product that may be evaluated in this article, or claim that may be made by its manufacturer, is not guaranteed or endorsed by the publisher.
