# Supplementary figures and images for: The cancer-associated fibroblast-related signature predicts prognosis and indicates immune microenvironment infiltration in gastric cancer
Source: Front Immunol. 2022 Jul 29;13:951214. doi: 10.3389/fimmu.2022.951214 (PMC9372353; doi:10.3389/fimmu.2022.951214)

## Supplementary Figures

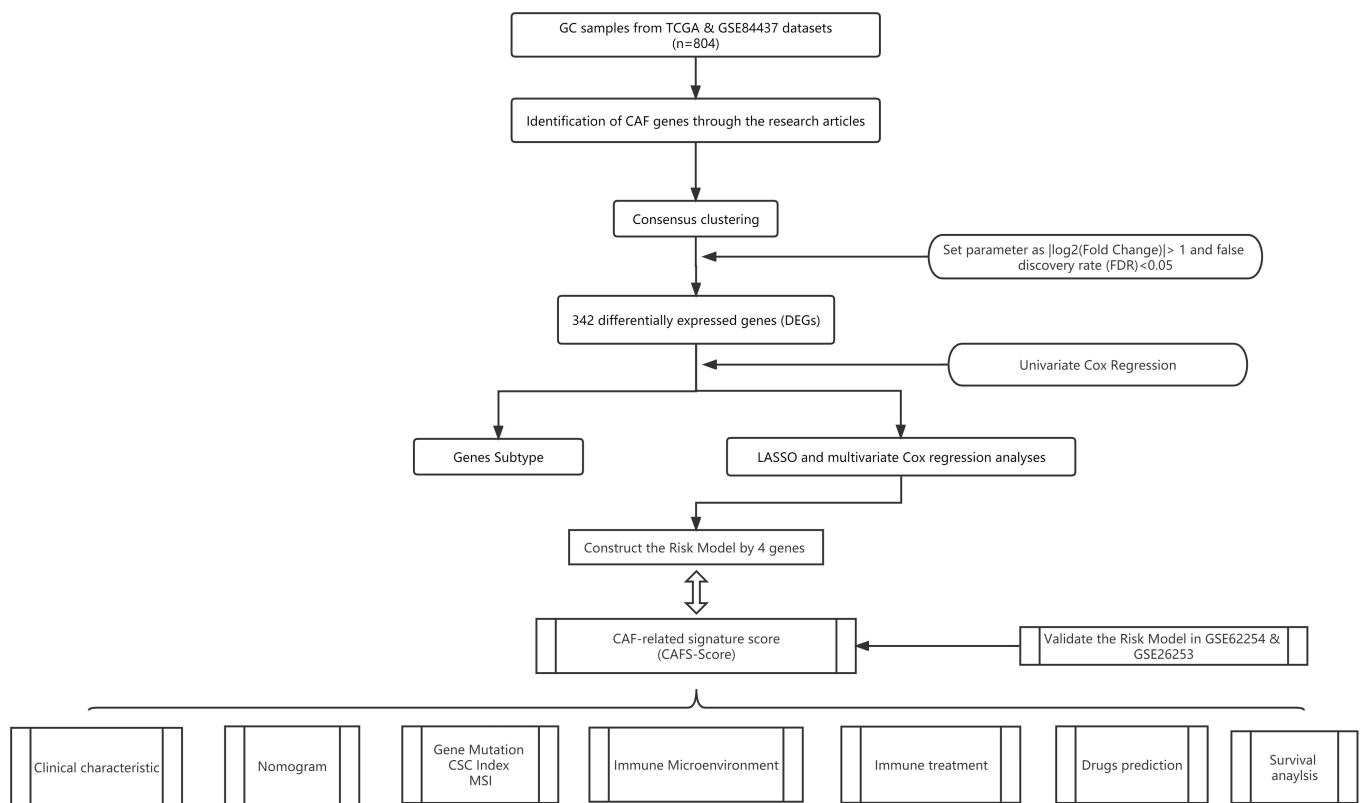

Supplementary Figure 1. The flow chat of the study.

Supplement: Supplementary file 1 [file DataSheet_1.pdf]
