# Supplementary material for: The cancer-associated fibroblast-related signature predicts prognosis and indicates immune microenvironment infiltration in gastric cancer
Source: Front Immunol. 2022 Jul 29;13:951214. doi: 10.3389/fimmu.2022.951214 (PMC9372353; doi:10.3389/fimmu.2022.951214)
Supplement: Supplementary file 2 [file DataSheet_2.pdf]

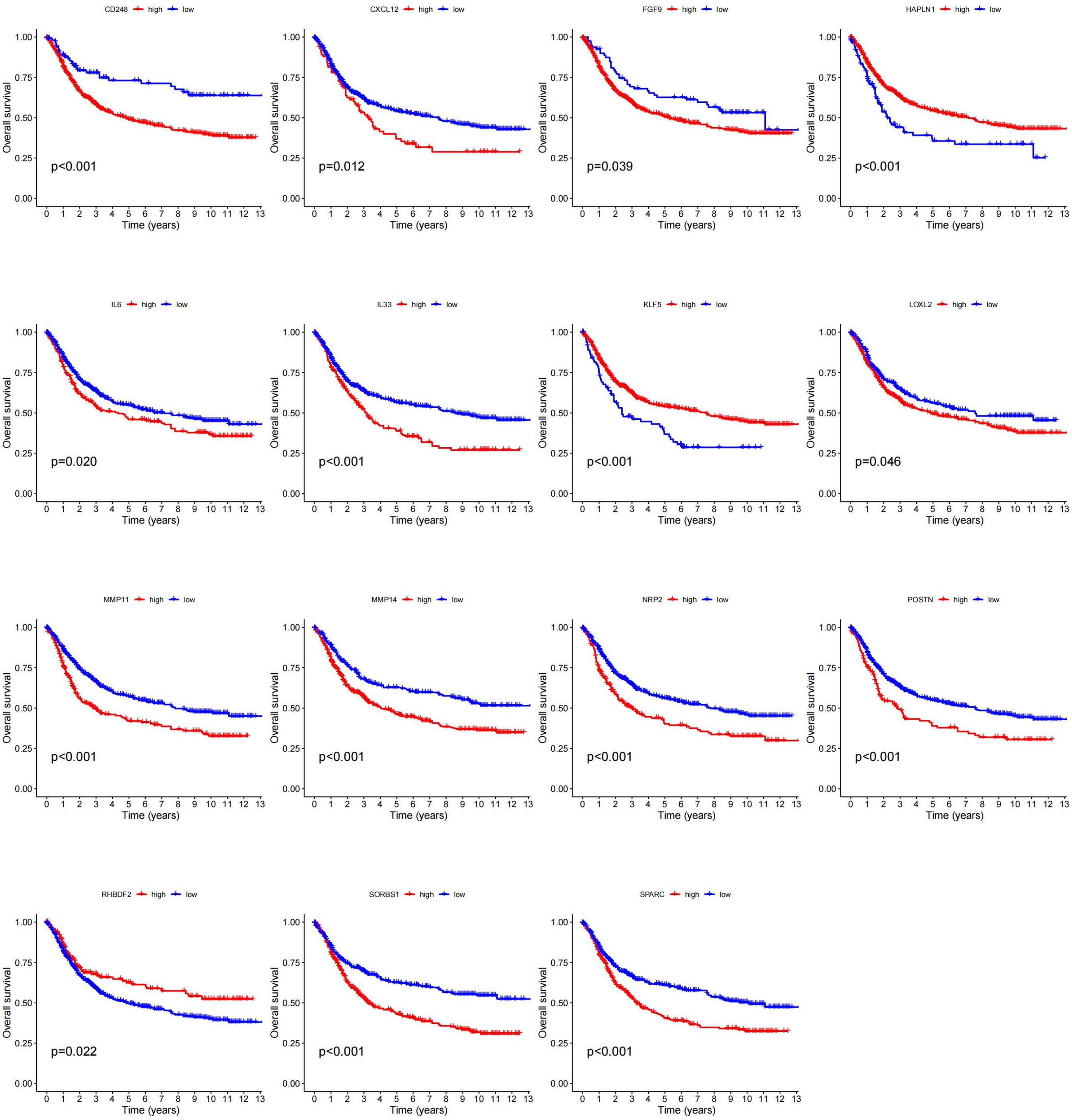

Supplementary Figure 2. The univariate Cox regression and Kaplan-Meier analysis of selected CAFs genes in 808 GC patients with statistically significant. CAFs, cancer-associated-fibroblasts.
