# Supplementary material for: The cancer-associated fibroblast-related signature predicts prognosis and indicates immune microenvironment infiltration in gastric cancer
Source: Front Immunol. 2022 Jul 29;13:951214. doi: 10.3389/fimmu.2022.951214 (PMC9372353; doi:10.3389/fimmu.2022.951214)
Supplement: Supplementary file 3 [file DataSheet_3.pdf]

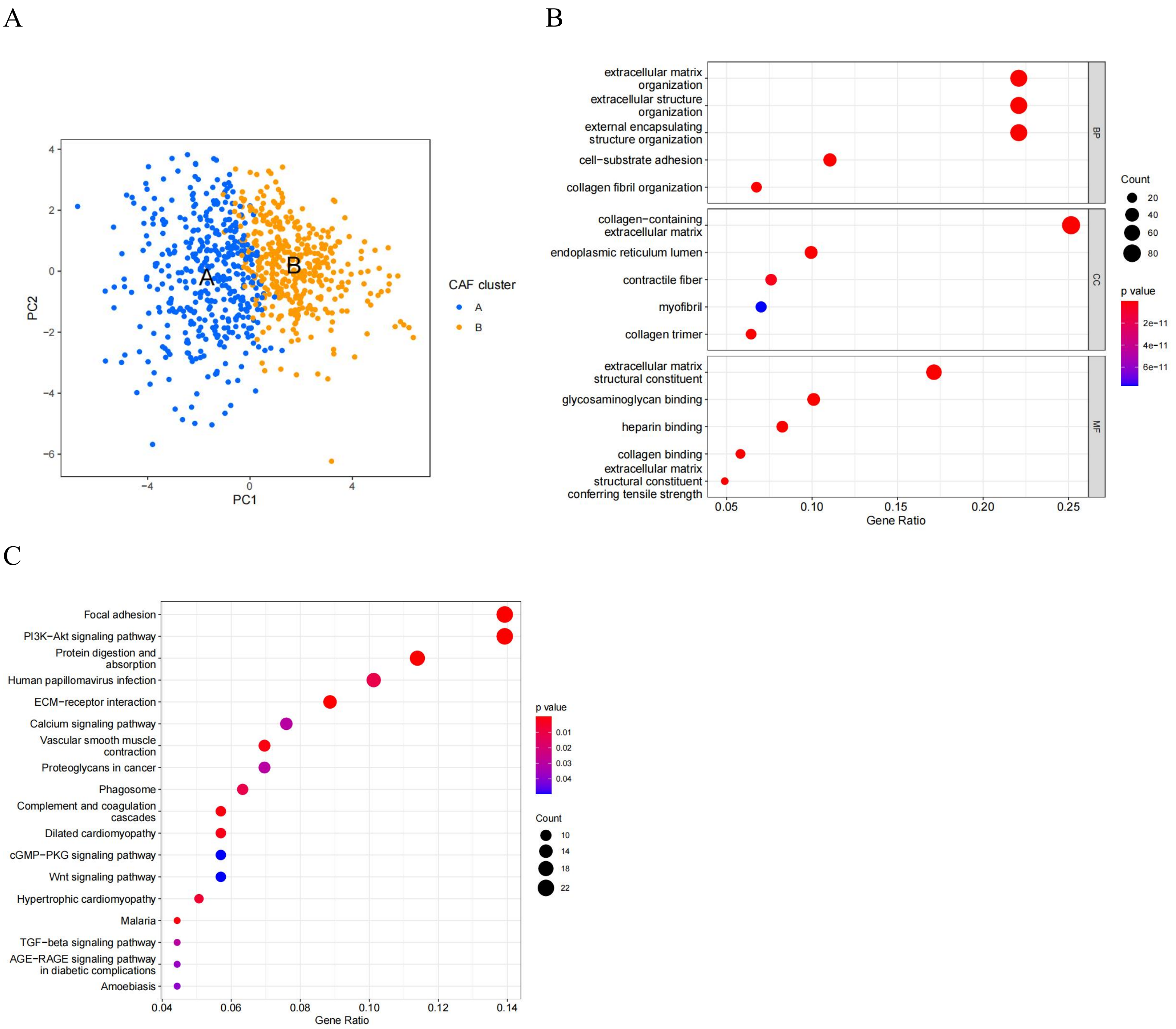

Supplementary Figure 3. CAF subtypes and biological characteristics of the DEGs. (A) PCA analysis representing a remarkable difference in transcriptomes between the two subtypes. (B-C) GO and KEGG enrichment analyses of DEGs among two CAF subtypes. DEGs, differentially expressed genes; CAFs, cancer-associated-fibroblasts; PCA, principal component analysis; Go, gene ontology; KEGG, Kyoto Encyclopedia of Genes and Genomes.
