# Supplementary material for: The cancer-associated fibroblast-related signature predicts prognosis and indicates immune microenvironment infiltration in gastric cancer
Source: Front Immunol. 2022 Jul 29;13:951214. doi: 10.3389/fimmu.2022.951214 (PMC9372353; doi:10.3389/fimmu.2022.951214)
Supplement: Supplementary file 4 [file DataSheet_4.pdf]

A B

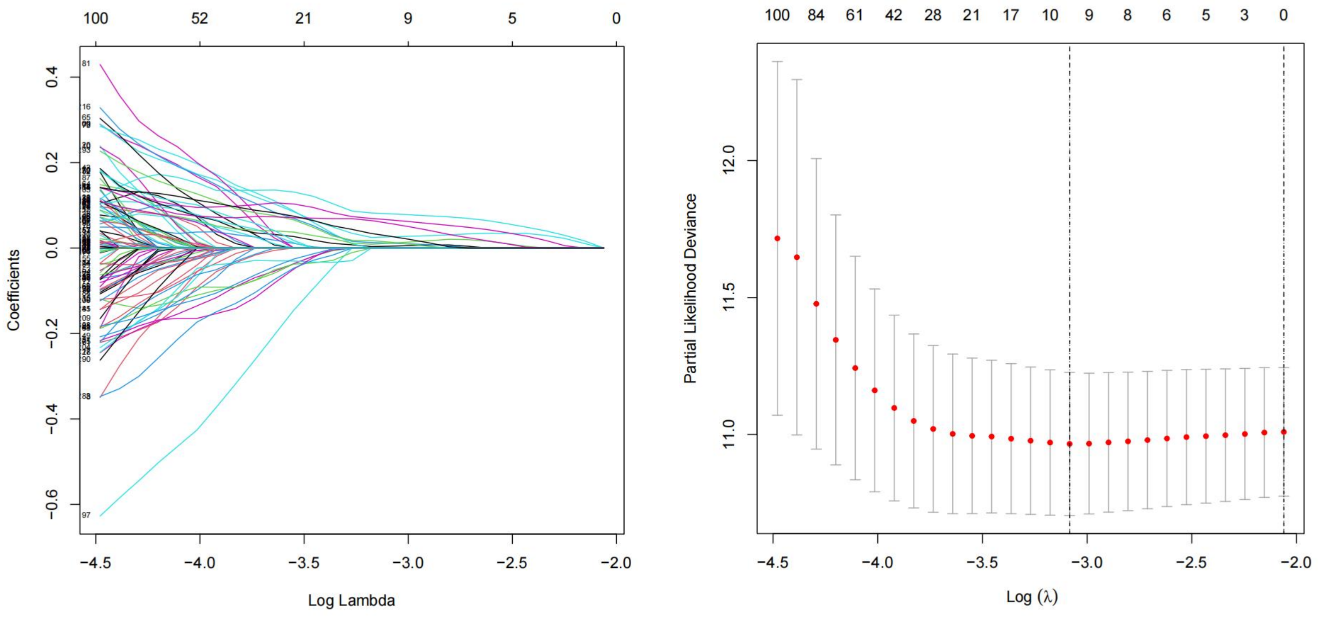

Supplementary Figure 4. Identifying representative candidate prognostic genes. (A-B) The LASSO regression analysis and partial likelihood deviance on the prognostic genes.
