# Supplementary material for: The cancer-associated fibroblast-related signature predicts prognosis and indicates immune microenvironment infiltration in gastric cancer
Source: Front Immunol. 2022 Jul 29;13:951214. doi: 10.3389/fimmu.2022.951214 (PMC9372353; doi:10.3389/fimmu.2022.951214)
Supplement: Supplementary file 5 [file DataSheet_5.pdf]

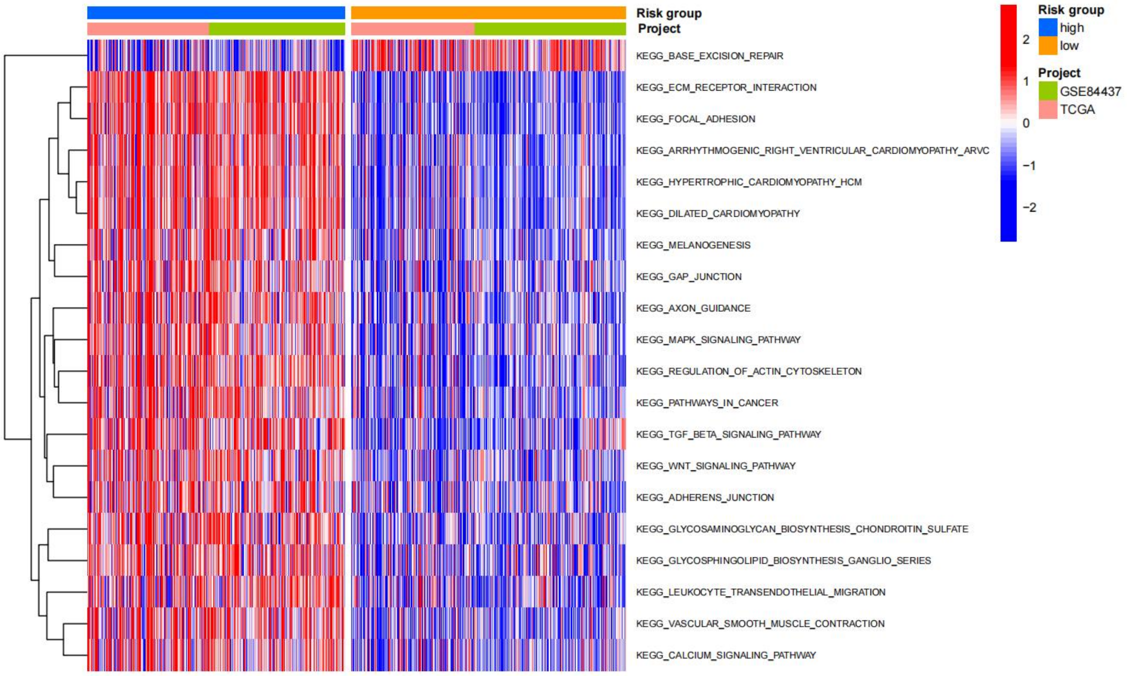

Supplementary Figure 5. GSVA enrichment analysis between the different risk subgroups. GSVA, gene set variation analysis.
