# Supplementary material for: The cancer-associated fibroblast-related signature predicts prognosis and indicates immune microenvironment infiltration in gastric cancer
Source: Front Immunol. 2022 Jul 29;13:951214. doi: 10.3389/fimmu.2022.951214 (PMC9372353; doi:10.3389/fimmu.2022.951214)
Supplement: Supplementary file 6 [file DataSheet_6.pdf]

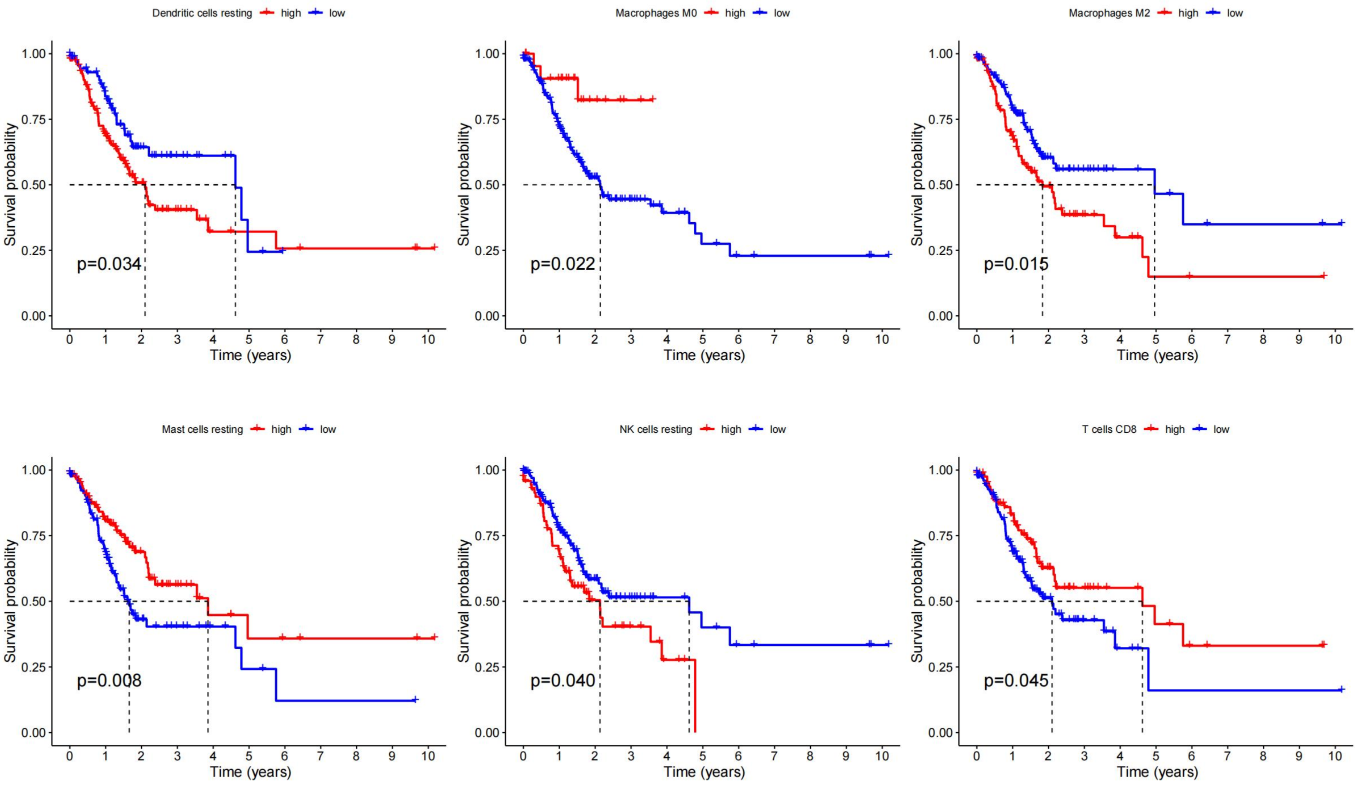

Supplementary Figure 6. The survival plots of different immune cells between high- and low-risk group with statistically significant.
