# Supplementary material for: The cancer-associated fibroblast-related signature predicts prognosis and indicates immune microenvironment infiltration in gastric cancer
Source: Front Immunol. 2022 Jul 29;13:951214. doi: 10.3389/fimmu.2022.951214 (PMC9372353; doi:10.3389/fimmu.2022.951214)
Supplement: Supplementary file 7 [file DataSheet_7.pdf]

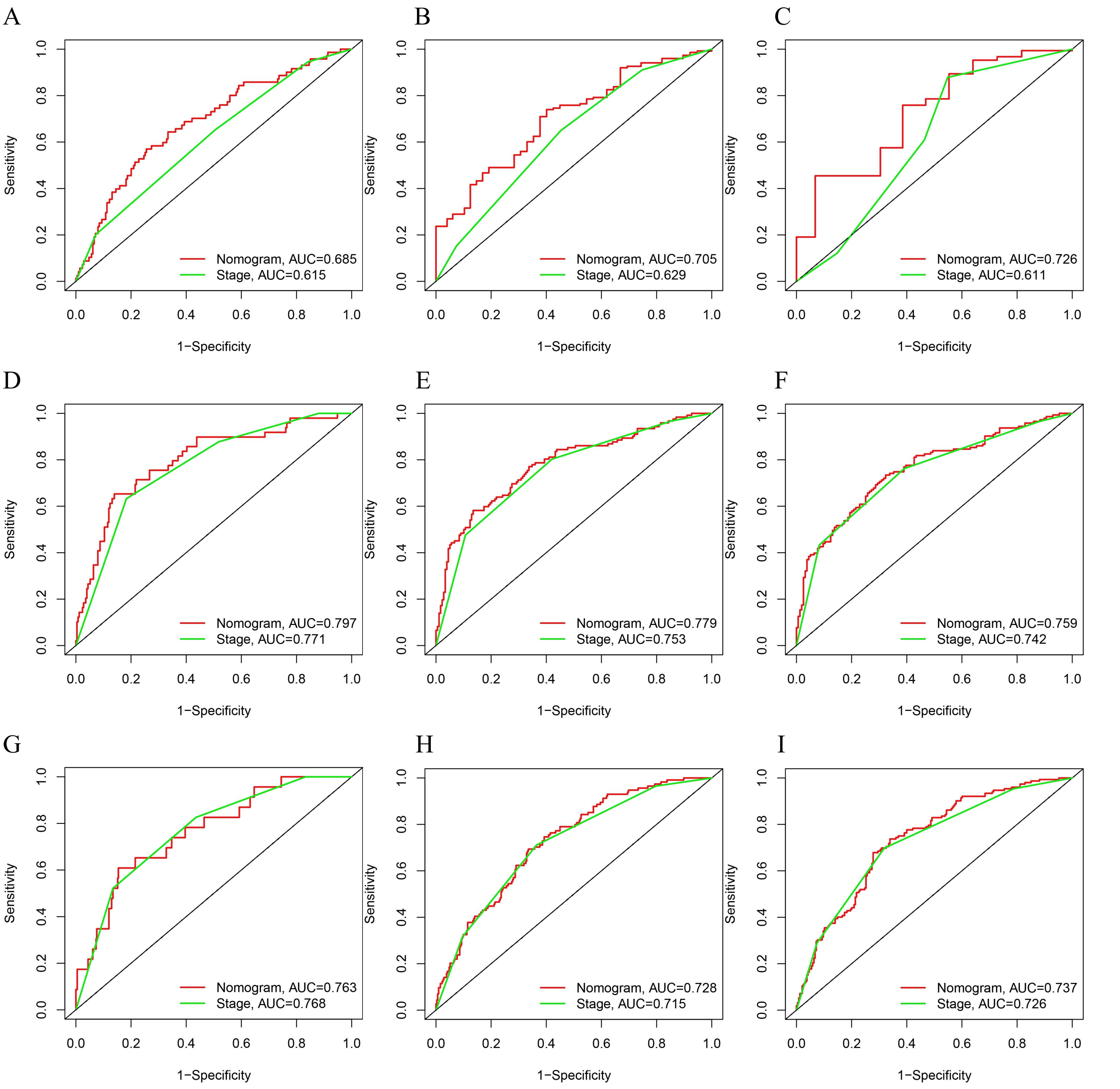

Supplementary Figure 7. Comparison of the AUCs of the nomogram and TNM staging system. (A-C) AUCs of the nomogram and TNM stage to predict overall survival at 1 years (A), 3 years (B), and 5 years (C) in TCGA cohort. (D-F) AUCs of the nomogram and TNM stage to predict overall survival at 1 years (D), 3 years (E), and 5 years (F) in GSE62254 cohort, respectively. (G-I) AUCs of the nomogram and TNM stage to predict overall survival at 1 years (G), 3 years (H), and 5 years (I) in GSE26253 cohort, respectively.
